# Comprehensive analysis of long non-coding RNA expression profiles in hepatitis B virus-related hepatocellular carcinoma

**DOI:** 10.18632/oncotarget.9880

**Published:** 2016-06-07

**Authors:** Xianli Gong, Wei Wei, Lan Chen, Zhi Xia, Chengbo Yu

**Affiliations:** ^1^ Department of Radiation Oncology, The First Affiliated Hospital, College of Medicine Zhejiang University, Hangzhou 310003, China; ^2^ State Key Laboratory for Diagnosis and Treatment of Infectious Diseases, The First Affiliated Hospital, School of Medicine Zhejiang University, Collaborative Innovation Center for Diagnosis and Treatment of Infectious Diseases, Hangzhou 310003, China

**Keywords:** hepatitis B virus, hepatocellular carcinoma, lncRNA, expression profile

## Abstract

Hepatocellular carcinoma (HCC) is one of the most common kinds of malignancies and is closely correlated with hepatitis B virus (HBV) infection. Recent evidence has proved that long non-coding RNAs (lncRNAs) are implicated in development and progression of cancer. However, the contributions of lncRNAs to HBV-related HCC remain largely unknown. Here, we comprehensively investigated lncRNA expression profiles in HBV-related HCC by annotating and analyzing microarray datasets. By analyzing 42 HCC tissue samples with different etiology (HBV-related, alcohol-related, and primary HCC) and 15 normal liver tissues, we identified 182 lncRNAs that were specifically differentially expressed in HBV-related HCC, namely HBV-related HCC specific lncRNAs(HH-lncRNAs). Using an online function annotation tool, we found these HH-lncRNAs were associated many oncogenes and immunity related biological processes. 6 candidate HH-lncRNAs were selected and further validated by quantitative real-time PCR analysis in a cohort of HCC tissue samples. Function of a candidate HH-lncRNAs, BAIAP2-AS1, was further predicted by co-expression network and gene set enrichment analysis. These findings provide insights into HH-lncRNAs and offer resource for further search of biomarkers and therapeutic targets of HBV-related HCC.

## INTRODUCTION

Hepatocellular carcinoma (HCC) is one of the most common malignant tumors and currently ranks the third course of cancer-related death [1]. Despite the treatment improvements achieved in surgical strategies and surveillance, the prognosis of HCC is still poor with a 5-year survival rate around 26% [2]. HCC is tightly correlated with hepatitis B virus (HBV) infection and almost half of HCC cases are associated with HBV infection. The geographic distribution of chronic HBV infection rates and HCC are highly similar [3]. However, the exact mechanism of HBV and HCC has not yet been clarified, herein, understanding mechanism of HBV in HCC development is crucial for early screening, clinical diagnosis, prevention, and prognosis of HCC patients.

The human genome contains about 20000 protein-coding genes, which account for only less than 2% of genome sequence [4]. However, about 90% of human genome is actively transcribed, yielding an extraordinary amount of RNA transcripts without protein-coding capacity [5]. Long non-coding RNA (lncRNA) is an important kind of non-coding RNA transcripts that range from 200nt to 100kb without protein-coding capacity [6, 7]. Recent years, increasing evidence have reported that lncRNAs participate in various biological processes [8, 9] and pathogenesis [10], especially tumorigenesis [11–13]. For HCC, lncRNA is involved in the HCC pathological processes by multiple mechanisms, including epigenetic silencing, mRNA splicing, and genetic variation [14–16]. For example, a novel lncRNA, HULC is upregulated in HCC compared with normal tissues and the expression level of HULC is correlated HBV infection status [17]. Additionally, HULC could potentially be a biomarker since HULC can be detected in both blood and tumor tissues of HCC patients [18].

The molecular mechanism underlying HBV-related HCC is still elusive and lncRNA may provide new insights into the underlying mechanism. On the other hand, few studies have characterized lncRNA expression profile in HBV-related HCC and the exact role of lncRNA in HBV-related HCC has not been clearly certified. Thus, we comprehensively investigated lncRNA expression profile in HBV-related HCC and tried to identify HBV-related HCC specific lncRNAs (HH-lncRNAs). In this study, we analyzed lncRNA expression profile in 57 tissue samples, including primary HCC, alcohol induced HCC, and HBV-related HCC, and normal liver tissues. A HH-lncRNA, BAIAP2-AS1 was characterized and we further predicted BAIAP2-AS1 function by co-expression network and potential competing endogenous RNA (ceRNA) relationship.

## RESULTS

### Dataset selection and probe annotation

**Figure 1:**
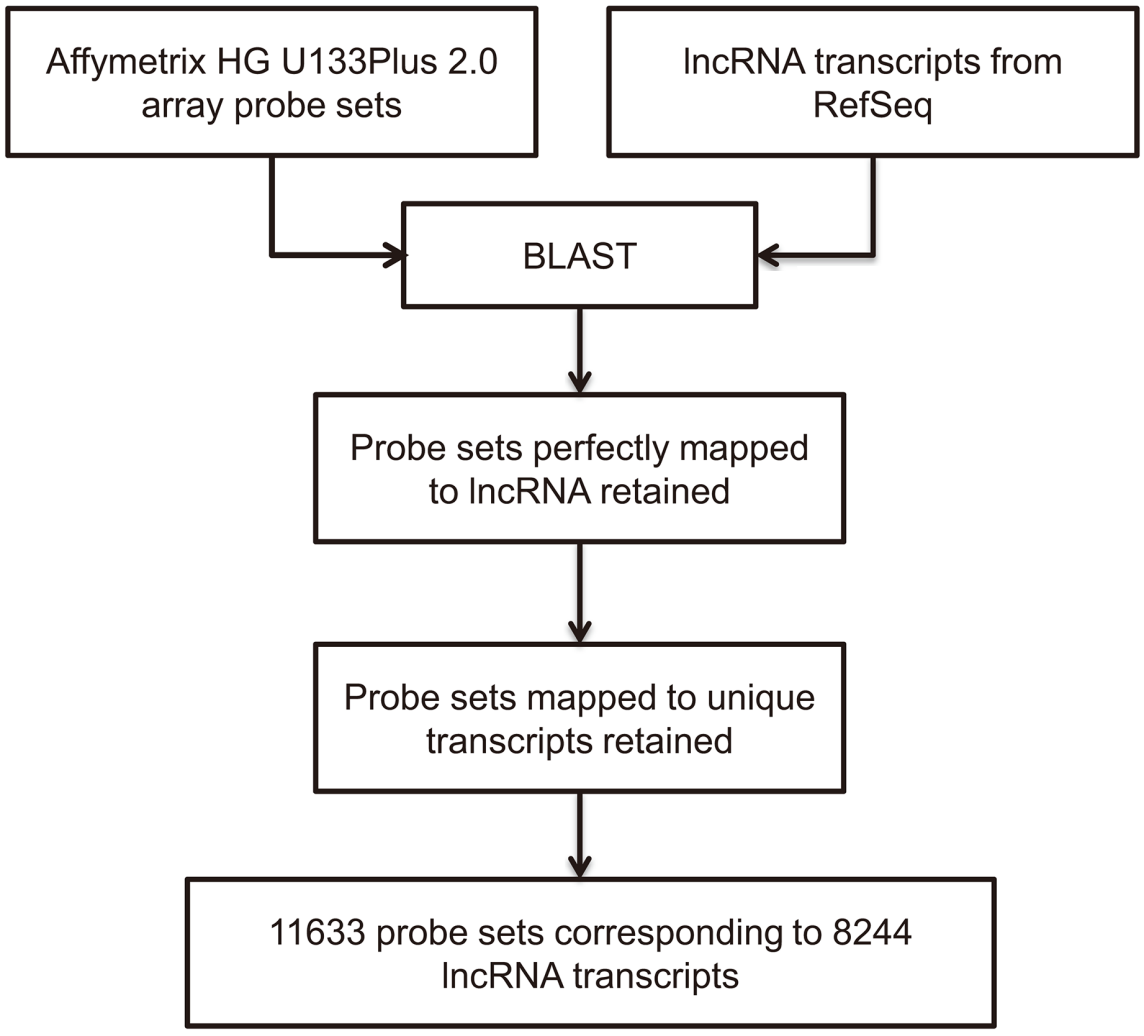
Computational pipeline for re-annotating the probes of the Affymetrix Human Genome U133 plus2.0 array

The Gene Expression Omnibus (GEO) is a public online database of high-throughput datasets, such as sequencing and microarray datasets. Profiling lncRNAs expression by annotation of microarray probe sets is a feasible and effective method, which has been utilized by many researchers [19, 20]. We searched GEO to identify datasets that included HBV-related HCC samples. The GSE62232 dataset met our criteria and the Affymetrix human genome U133 (HG U133) Plus2.0 microarray platform was used in this study. Probe sets of HG U133 Plus2.0 microarray were annotated by blasting with probe sequences with lncRNA transcripts from RefSeq database (Figure 1. Probe sets annotation pipeline). 8244 lncRNA transcripts were annotated in the HG U133 Plus2.0 microarray.

### Differential expression of lncRNAs in HBV-related HCC

**Figure 2:**
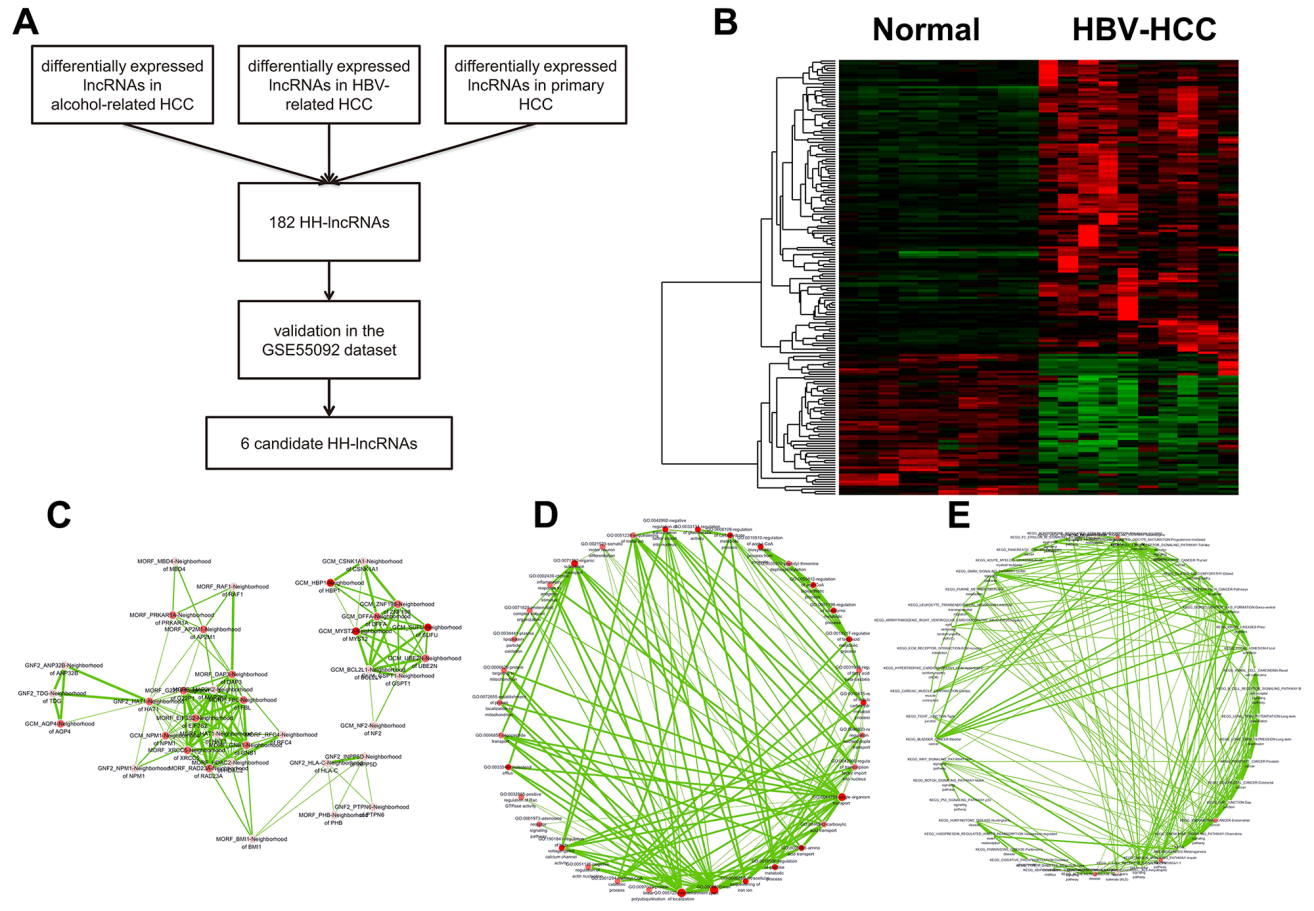
**A.** Flow chart of data analyses. **B.** Heatmap of 182 HH-lncRNAs in the GSE62232 dataset. **C.** Neighbor genes of the 182 HH-lncRNAs. GO items **D.** and KEGG pathways **E.** enriched among the neighbor genes of HH-lncRNAs

In the GSE62232 dataset, 4 groups of patient samples were retrieved and analyzed: normal liver tissues (n=10), HBV-related HCC (n=10), alcohol-related HCC (n=22), and primary HCC (n=15). As shown in Figure 2A, primary and alcohol-related HCC were selected as control groups to identify HH-lncRNAs. 182 HH-lncRNA transcripts were achieved in this way (Figure 2B and Supplementary Table S1).

Although many cancer-associated lncRNAs have been characterized, it is hard to predict function and potential molecular mechanism of lncRNAs. Genomic location may provide insights into potential function of lncRNAs since many lncRNAs exert their function *in cis*. To probe the potential biological function of the 182 lncRNAs we identified, the online annotation tool, GREAT (genomic regions enrichment of annotations tool) was used, which annotates genomic regions according to neighboring genes [21]. Predicted by GREAT, HH-lncRNAs were located around many pro-oncogenes, like HBP1, WNT1, and MMP1 (Figure 2C). As shown, immune system associated GO items (regulation of CD8-positive, alpha-beta T cell differentiation) and KEGG pathways (Primary immunodeficiency) were enriched in the 182 HH-lncRNAs (Figure 2D, 2E), indicating these HH-lncRNAs may play important roles in the pathologic process of HBV-related HCC. Functional annotation results of GREAT were shown in Supplementary Table S2.

### Characterization of lncRNA BAIAP2-AS1

**Figure 3:**
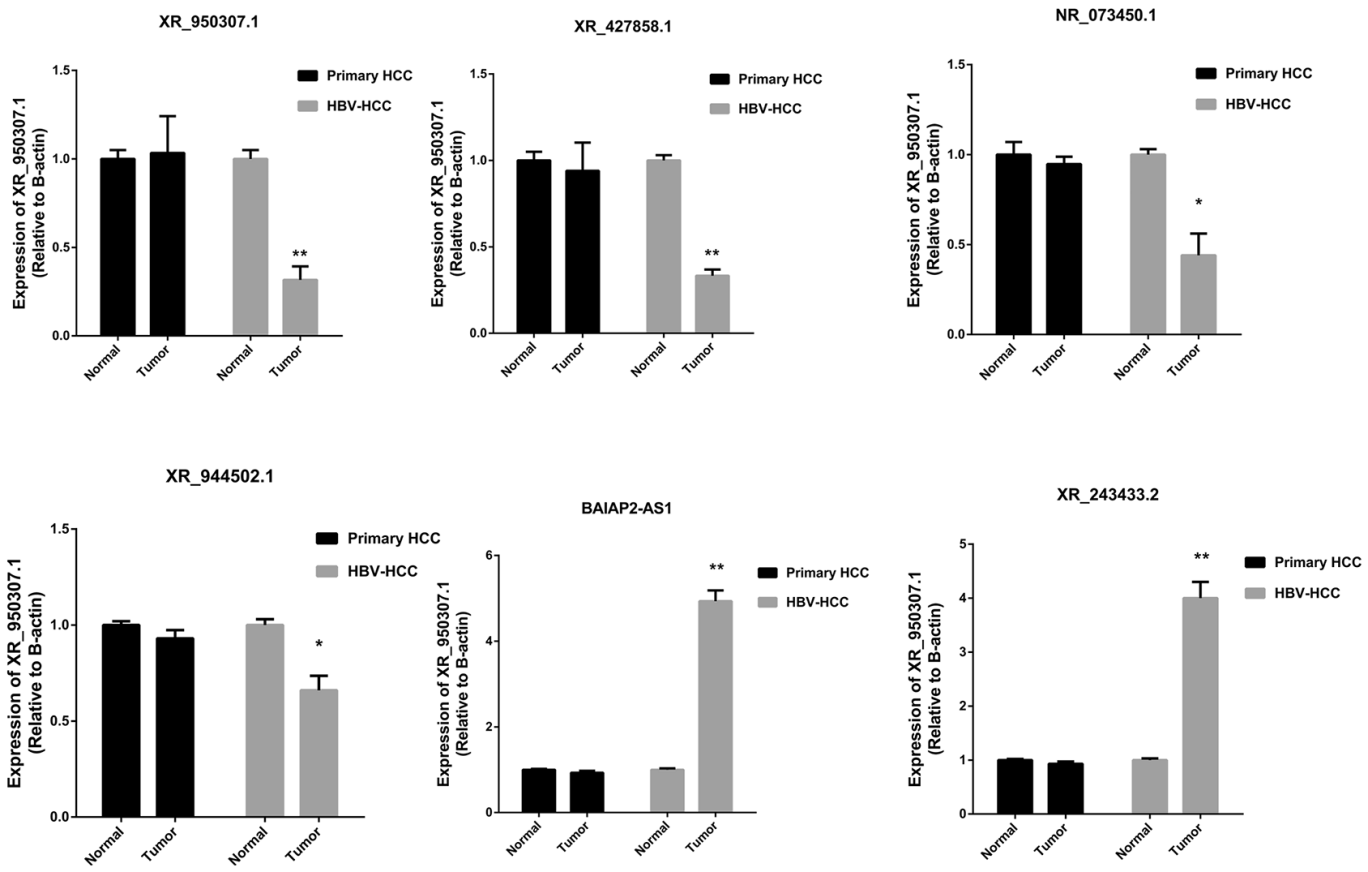
Expression of 6 HH-lncRNAs was validated among patients with primary HCC and HBV-related HCC *P <0.05, **<P<0.01.

We then tried to identify candidate HH-lncRNAs among the 182 differentially expressed lncRNAs. Thus, we validated the 182 HH-lncRNAs in the GSE55092 dataset, in which tissue samples were laser capture-microdissected to avoid contamination of non-cancerous cells. Thus, 6 HH-lncRNAs were filtered out (Table 1) in this way. To validate expression of the 6 HH-lncRNAs, qRT-PCR was performed in a cohort of 20 HCC patients, including 10 primary HCC and 10 HBV-related HCC. As shown in Figure 3, expression of 6 HH-lncRNAs validated by qRT-PCR was consistent with microarray results. No difference was found between primary HCC and paired normal tissues, while they were significantly differentially expressed between HBV-related HCC and paired normal tissues. Yu TT has analyzed lncRNA expression in HBV-related HCC tissues with microarray [22], and found a significant up-regualted lncRNA, ULK4P2. However, ULK4P2 was not found in our results, which may be explained by different annotation methods. QRT-PCR analysis found that ULK4P2 was up-regualted in both primary HCC and HBV-related HCC tissues (Supplementary Figure S1).

**Table 1:** Characteristics of the 6 candidate HH-lncRNAs

Refseq Accession	logFC	chr	start	end	strand	Description
XR_950307.1	−1.94	chr11	75205717	75225564	-	Homo sapiens uncharacterized LOC105369388 (LOC105369388), transcript variant X4, ncRNA
XR_427858.1	−1.7	chr6	46652945	46678190	+	Homo sapiens solute carrier family 25, member 27 (SLC25A27), transcript variant X1, misc_RNA
NR_073450.1	−1.38	chrX	54933134	54994078	-	Homo sapiens 6-phosphofructo-2-kinase/fructose-2,6-biphosphatase 1 (PFKFB1), transcript variant 4, non-coding RNA
XR_944502.1	−1.05	chr12	128853440	128984962	+	Homo sapiens glycosyltransferase 1 domain containing 1 (GLT1D1), transcript variant 2, non-coding RNA
NR_026857.1	1.02	chr17	81029133	81034719	-	Homo sapiens BAIAP2 antisense RNA 1 (head to head) (BAIAP2-AS1), long non-coding RNA
XR_243433.2	1.05	chr16	68263830	68301819	+	Homo sapiens solute carrier family 7 (amino acid transporter light chain, y+L system), member 6 (SLC7A6), transcript variant X7, misc_RNA

Among 6 candidate HH-lncRNAs, BAIAP2-AS1 was selected for further characterization, since it is mostly up-regualted. BAIAP2-AS1 is a heat-to-head antisense lncRNA of BAIAP2. The BAIAP2-AS1 gene is located in the genomic region of chr17q25.3, encoded a 4421nt RNA transcript. Some antisense lncRNAs may affect expression of their neighbor coding genes [23, 24]. However, we found no significant association between BAIAP2-AS1 and BAIAP2 expression, indicating BAIAP2-AS1 possibly function independent of BAIAP2.

**Figure 4:**
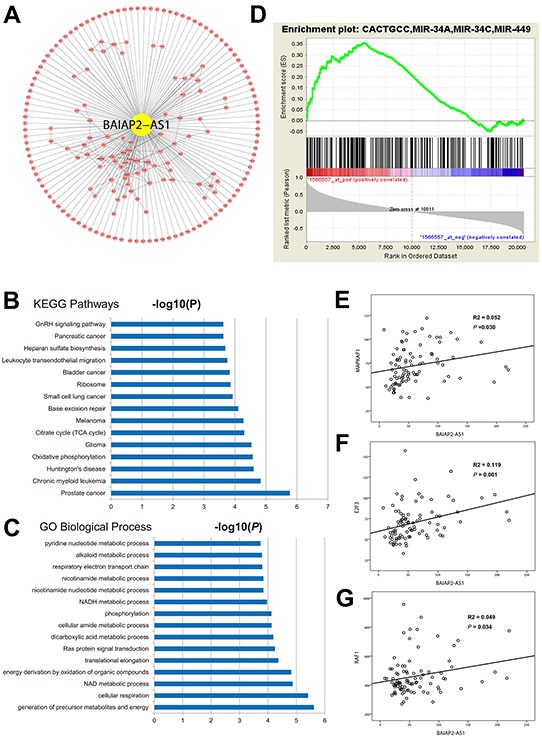
Co-expression network of BAIAP2-AS1 in the TCGA HCC dataset **A.** KEGG pathways and GO items enriched among genes co-expressed with BAIAP2-AS1 in the TCGA HCC dataset **B, C.** GSEA results showed that target genes of miR-34A were positively correlated with BAIAP2-AS1 **D.** Positively correlation between BAIAP2-AS1 and MAPK1, E2F3, and RAF1 in the GSE62232 dataset **E, F, G.**

It is widely accepted that genes regualted by the same regulator or a set of genes with the same function would be co-expressed and co-expression network has been used to predict function of unknown genes [25]. Genes that are co-expressed with BAIAP2-AS1 in human live cancer are retrieved through the online database of the cancer genome atlas (TCGA) data (methods section). We therefore constructed co-expression network for BAIAP2-AS1 (Figure 4A). Functional annotation analyses shown that metabolism related GO items, and cancer associated KEGG pathways were enriched among the genes co-expressed with BAIAP2-AS1 (Figure 4B, 4C). Pandolfi PP has proposed the competing endogenous RNA (ceRNA) hypothesis in 2011 [26] and many lncRNAs has confirmed the ceRNA mechanism of lncRNAs [27]. According to ceRNA hypothesis, RNA transcripts with ceRNA relationship share the same miRNA biding sites and their expression levels are positively correlated. Thus, we hypothesized that BAIAP2-AS1 may function as ceRNA. By online tool, we found several conserved miRNA binding sites in the sequence of BIAPA2-AS1 (Supplementary Table S3). Gene set enrichment analyses (GSEA) showed target mRNAs of miR-491, miR-331, and miR-34A were positively enriched with BAIP2-AS1 (Figure 4D), namely, these mRNAs were positively correlated BAIAP2-AS1 expression. In addition, BAIAP2-AS1 also harbors binding sites of miR-491, miR-331, and miR-34A. We then analyzed the expression correlation in the GSE62232 dataset. As expected, BAIAP2-AS1 is significantly positively correlated with MAPKAP1 (target of miR-491, coefficient=0.227, P=0.030), E2F3 (target of miR-34A, coefficient =0.345, P=0.001), and RAF1 (target of miR-331, coefficient =0.223, P=0.034) (Figure 4E–4G).

**Figure 5:**
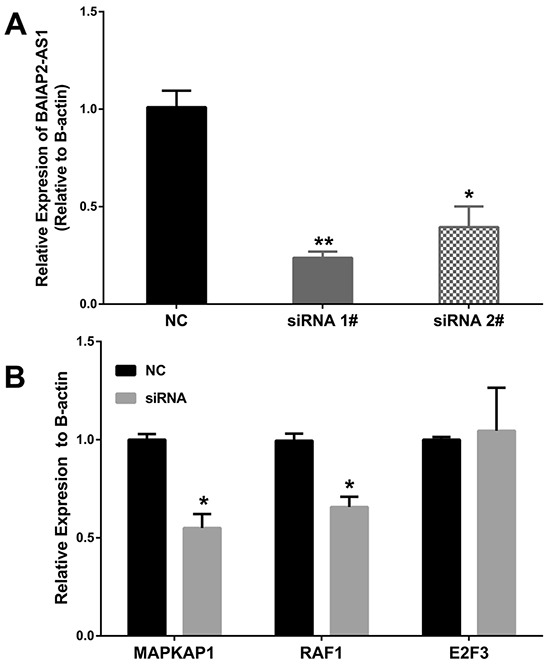
BAIAP2-AS1 was significantly silenced by siRNA 1# **A.** compared with negative control (NC). And after silence of BAIAP2-AS1, the expression levels of MAPKAP1 and RAF1 decreased, while E2F3 showed no significant differences **B.** *P<0.05, **<P<0.01.

To further investigate the ceRNA hypothesis of BAIAP2-AS1, 2 small interfering RNAs (siRNAs) specifically targeting BAIAP2-AS1 were synthesized and transfection of siRNA 1# significantly knockdown BAIAP2-AS1 expression in HepG2 cells (Figure 5A). Then, we analyzed MAPKAP1, E2F3, and RAF1 expression levels after BAIPA2-AS1 silence by qRT-PCR (Figure 5B), and the results showed that the expression of MAPKAP1 and RAF1 decreased, while E2F3 expression level showed no difference. These data suggested BAIAP2-AS1 may function as a ceRNA.

## DISCUSSION

In the current study we comprehensively analyzed lncRNA expression in HBV-related HCC and identified 182 HH-lncRNAs. Compared with previous reports [22], our study has several advantages. First, alcohol-related HCC and primary HCC were treated as control groups to identified lncRNAs that are specifically differentially expressed in HBV-related HCC. Second, functional annotation and prediction was performed for the 182 HH-lncRNAs and an individual HH-lncRNA, BAIAP2-AS1. Third, compared with other reports that compared HCC tissues to adjacent normal tissues, sample size was larger in our analyses, which might provide better statistical efficacy.

LncRNA has been thought as junk RNA and transcription noise, however, recent evidence have proved lncRNAs are involved in various biological and pathological processes [7, 12]. LncRNAs have been found to function at almost every aspect in regulation of gene: epigenetic, transcriptional, post-transcriptional, and translational. Due to the advance of high-throughput sequence and microarray of the transcriptome, thousands of lncRNAs have been discovered and are found differentially expressed. However, little is known about the possible biological functions. For example, in the aspect of HBV-related HCC, Yu and colleagues performed an lncRNA microarray study to analyze lncRNA expression but they did not characterize or predict function of a specific lncRNA [22]. In the current study, we identified 182 HH-lncRNAs and based on their genomic location, we predicted potential function of these lncRNAs with an online tool. As shown, several immune related items were enriched, such as T cell reporter signaling pathway, B cell receptor signaling pathway, and regulation of CD8-positive, alpha-beta T cell activation. These results indicated that the 182 HH-lncRNAs might be involved in these biological processes.

CeRNA hypothesis was proposed in 2011 and more and more evidence has supported this mechanism [26]. For example, the well-known lncRNA, HOTAIR, could function as a sponge of miR-331-3p to promote gastric cancer progression [28]. In this study, we find lncRNA BAIAP2-AS1 harbors several conserved miRNA binding sites and is possibly function as miRNA sponge to promote HBV-related HCC. We found BAIAP2-AS1 shares the same miRNA binding sites with E2F3, RAF1, and MAPK1, and BAIAP2-AS1 expression level was positively correlated with these genes. E2F3 is a member of the E2F transcription factors that are master regulators of many biological processes, like DNA repair, apoptosis, cell cycle, and centrosome duplication [29]. E2F3 is overexpressed in various cancers and drives tumorigenesis [30]. RAF1 is also a pro-oncogene and serves as a part of the mitogen-activated protein kinases/extracellular signal-regulated kinase signal transduction pathway and regulates cell migration, apoptosis, and differentiation. Raf1 phosphorylates and activates Raf-MEK-ERK pathway, which then regulates cell cycle, proliferation, apoptosis, and migration [31]. However, after silence of BAIAP2-AS1, expression level of E2F3 was not down-regualted. According to a recent review [32], the reason may be that miR-34A is an abundant miRNA and silence of BAIAP2-AS1 is not enough to affect miR-34A activity. Also, the endogenous target pool of miR-34a may also account for this. However, further experiments are needed to validate this hypothesis.

To summary, we comprehensively characterize lncRNA expression profile in HBV-related HCC and provide a basis for understanding roles of lncRNA in HBV-related HCC.

## MATERIALS AND METHODS

### Microarray dataset information

Gene expression data used in the current study was obtained from the publicly available Gene Expression Omnibus (GEO) database. The data sets (GSE62232 and GSE55092) were performed on the Affymetrix HG-U133 Plus 2.0 platform. The HG-U133 Plus 2.0 microarray is widely used to profile genome-wide gene expression, which proved abundant resource for data mining. The raw CEL files were downloaded and quantile normalized and background adjusted using Robust Multichip Average (RMA, Windows Version) [33, 34]. After normalization, expression value of each probe was obtained. The normalized data were then analyzed with linear models for microarray data (LIMMA) [35] using the Bioconductor package through R 3.0.1. The probe sets with adjusted *P* value below 0.05 and absolute fold change > 2 was defined as significantly different.

### Probe sets annotation

To identify the probe sets mapped to lncRNAs, we developed an lncRNA annotation pipeline. First, lncRNA transcripts were downloaded from the NCBI Refseq database and probe sequences of HG-U133Plus 2.0 microarray were also downloaded from the Affymetrix website. Then, the sequences of probe sets and sequences of lncRNAs were compared with BLAST software. Only sequences of a probe set were perfectly matched with an lncRNA, then the probe set was considered as matched with this lncRNA; otherwise, the BLAST result was abandoned. Thus, an lncRNA re-annotation pipeline was built and the differentially probe sets were filtered and annotated with this pipeline.

### Patients and tissue samples

This study was approved by the Ethics Committee of The First Affiliated Hospital, College of Medicine, Zhejiang University. Paired HCC tissues and adjacent normal tissues were obtained from 20 patients who received treatment in The First Affiliated Hospital between 2009 and 2014. All tissue samples were snap-frozen and stored at −80 until total RNA extraction. All tumor and paired normal tissues were confirmed by experienced pathologists. Informed written consents were obtained from all patients included in this study.

### Cell culture and transfection

HepG2 cells were purchased from ATCC and maintained in DMEM supplemented with 10% FBS. Cells were cultured at 37°C in a humidified atmosphere containing 95% air and 5% CO2.

Small interfering RNA (siRNA) specific for BAIAP2-AS1 (siRNA 1# and siRNA 2#) and negative control was synthesized (GenePharm, Shanghai, China) and transfected using Lipofectamine 2000 in HepG2 cells according manufacture instruction. The sequences of si-BAIAP2-AS1 were: siRNA 1#: GCAGGCATGGTGTGCATTT; siRNA 2#: GCACCTGAGAGGTGATCAT.

### RNA extraction and qRT-PCR analysis

RNA of tissue sample was isolated with TRIzol reagent (Invitrogen, Carlsbad, CA, USA) according to the manufacture's protocol. 1000 ng total RNA was reversely transcribed into a final volume of 20 μl using the PrimerScript RT Master Mix (Takara, cat: RR036A). The quantitative real-time polymerase chain reaction (qRT-PCR) was performed using the SYBR Select Master Mix (Applied Biosystems, cat: 4472908) on ABI 7500 system (Applied Biosystems, Foster City, CA, USA) according to the manufacturer's instructions. B-actin was measured as an internal control paired tumor and normal tissues. After the reverse transcription, 0.5 μl of the complementary DNA was used for subsequent qRT-PCR reaction. The PCR primers used were provided in Supplementary Table S4. The −ΔΔCt-method was used to measure expression level of target genes.

### Statistical and bioinformatics analyses

GREAT analyses were performed by the website (http://bejerano.stanford.edu/great/public/html/). Gene Ontology (GO) and KEGG pathway analyses were conducted using DAVID website (https://david.ncifcrf.gov/home.jsp). GSEA was performed by the GSEA software and gene sets used in this work were downloaded from the Molecular Signatures Database (http://software.broadinstitute.org/gsea/msigdb/index.jsp, MSigDB v4.0, released Jun 7, 2013). According to BAIAP2-AS1 expression, samples were classified into 2 groups: high expression and low expression. Genes co-expressed with BAIAP2-AS1 in HCC was obtained from the online database (http://lncrnator.ewha.ac.kr), which collected TCGA data. Co-expression network was constructed by Cytoscape software. Paired T test were used to analyze PCR results and P<0.05 was considered statistically significant.

## SUPPLEMENTARY FIGURE AND TABLES










