# Supplementary material for: Comprehensive analysis of long non-coding RNA expression profiles in hepatitis B virus-related hepatocellular carcinoma
Source: Oncotarget. 2016 Jun 7;7(27):42422–30. doi: 10.18632/oncotarget.9880 (PMC5173145; doi:10.18632/oncotarget.9880)
Supplement: Supplementary file 1 [file oncotarget-07-42422-s001.pdf]

Comprehensive analysis of long non-coding RNA expression profiles in hepatitis B virus-related hepatocellular carcinoma

SUPPLEMENTARY FIGURE AND TABLES

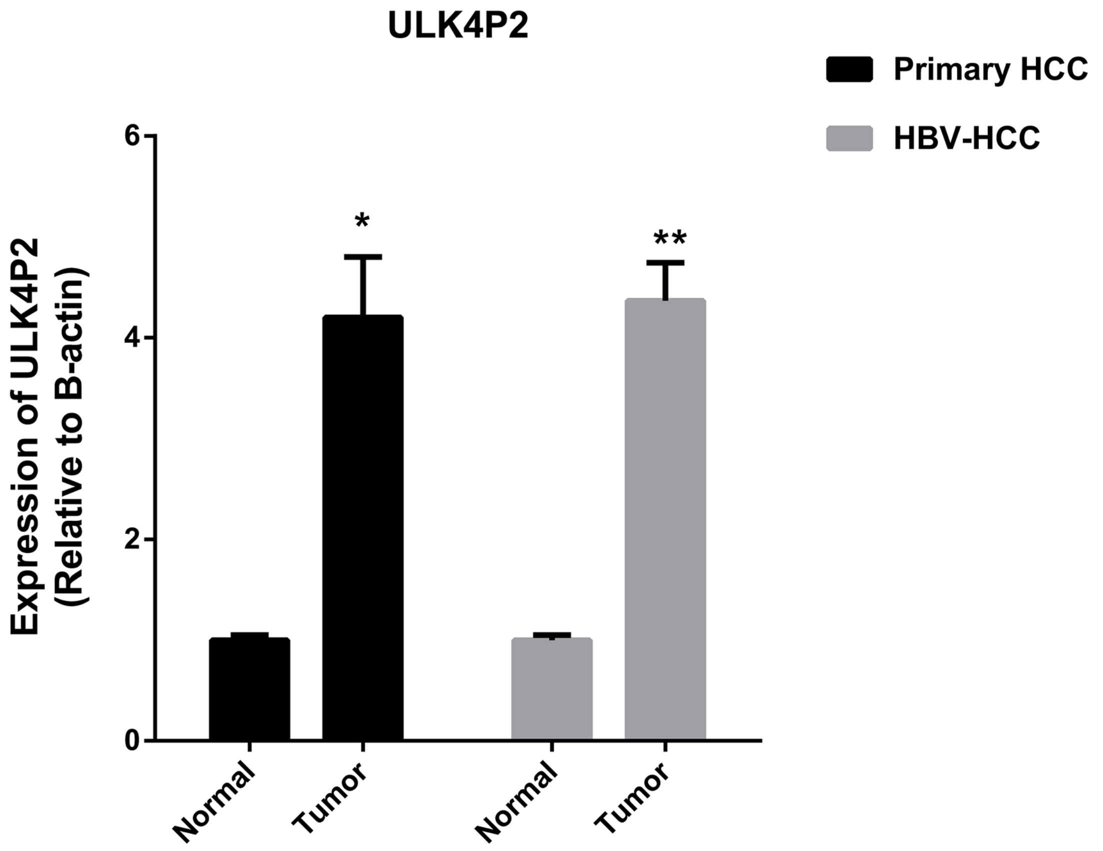

Supplementary Figure S1: Expression of ULK4P2 is up-regualted in both primary HCC and HBV-related HCC tissues. *P<0.05; **P<0.01.

Supplementary Table S1: 182 HH-lncRNAs.

See Supplementary File 1

Supplementary Table S2: GREAT results of the 182 HH-lncRNAs.

See Supplementary File 2

Supplementary Table S3: Predicted miRNA binding sites in the sequence of BAIAP2-AS1.

See Supplementary File 3

Supplementary Table S4: PCR primers.

See Supplementary File 4
